# Estimating the distribution of reedbed in Britain demonstrates challenges of remotely sensing rare land cover types at large spatial scales

**DOI:** 10.1038/s41598-024-73030-6

**Published:** 2024-09-27

**Authors:** Jacob G. Davies, Calvin Dytham, Robert A. Robinson, Colin M. Beale

**Affiliations:** 1 Department of Biology, University of York, Wentworth Way, York, YO10 5DD UK; 2 British Trust for Ornithology Scotland, Beta Centre (Unit 15), Stirling University Innovation Park, Stirling, FK9 4NF UK; 3 British Trust for Ornithology, The Nunnery, Thetford, Norfolk, IP24 2PU UK; 4 York Environmental Sustainability Institute, University of York, Wentworth Way, York, YO10 5DD UK; 5 Leverhulme Centre for Anthropocene Biodiversity, University of York, York, YO10 5DD UK

**Keywords:** Wetlands ecology, Conservation biology

## Abstract

Common Reed *Phragmites australis*, globally one of the mostly widely distributed wetland plants, is important for biodiversity and for humans. However, like most wetland plant communities, reedbed has rarely been mapped at large geographical scales, restricting the information available to study reed’s range dynamics or inform its management. Using Sentinel-2 data and machine learning, we aimed to produce the first published remotely-sensed reedbed map of Britain; however, accuracy as assessed by field validation was relatively low (AUC = 0.671), with many false positives (commission error of 93.4%). A similar workflow carried out in Google Earth Engine, using nearly an order of magnitude more images, gave a lower commission error but a disproportionately higher omission error. Using the known commission and omission error, we estimate that in 2015–2017 ~ 7800 ha of Britain was reedbed. Our study highlights the enduring barriers to accurate land cover classification at large spatial scales. Even with a ‘big data’ approach, reflectance error and ecological factors such as confusion land cover types and geographical variation in temporal reflectance function will probably continue to limit the size of area for which land cover can be classified accurately, therefore limiting the utility of remote sensing for ecologists.

**Supplementary Information:**

The online version contains supplementary material available at 10.1038/s41598-024-73030-6.

## Introduction

Global wetland area is rapidly declining, with a net loss of 21% over the last three centuries^1^, causing declines in biodiversity and ecosystem services^2^. However, quantitative estimates of wetland area at national scales are scarce^3^, or are derived from incomplete inventories of individual wetlands^4^. To inform policy, for example the initiative to conserve or restore 30% of Earth’s ecosystems by 2030^5^, there is a need for wetland inventories on a larger geographical scale^6^.

Common Reed *Phragmites australis* (hereafter ‘Reed’) is a perennial, wind-pollinated helophytic grass which produces annual shoots of up to 5.3 m in height^7^. Reed is one of the most widely distributed wetland plants globally, found on all continents except Antarctica^7^. Due to its highly competitive ability in specific environmental conditions, it commonly forms large dominant or monodominant patches (hereafter ‘reedbed’). Reed is globally important for biodiversity, ecosystem function, nutrient cycling, and as a resource for humans. Some species (e.g. Eurasian bittern *Botauris stellaris*, southern wainscot *Mythimna straminea*) are almost entirely restricted to reedbeds. Reed expedites ecological succession from open water to land, plays a complex role as a greenhouse gas source and sink^8^, and is widely used by humans^9^: for example, for water treatment, biofuel and thatch. In the last century, Reed has colonised large areas outside of its native range^10^, influencing invasion biology^11^. Due to its invasiveness and status as an agricultural weed, major efforts have been made to eradicate Reed outside of its native range^12^. Reed is not globally under threat^13^, but has a dynamic geographical range^14^, and so reedbed is often a conservation priority at national scales^15–17^. To understand its range dynamics and to target its conservation or management, it is important to map reedbed distribution at large spatial scales and develop tools to allow monitoring of reedbed distribution over time.

Plant species vary in electromagnetically distinctive characteristics (e.g. concentration of photosynthetic pigments), which themselves vary through the year, and thus can often be distinguished remotely, through classification of spectral reflectances. Furthermore, the training of classification algorithms is aided by Reed’s frequent monodominance, easing its identification. Additionally, fieldwork for the mapping of wetland vegetation is expensive and time-consuming, and can be limited in extent by cost and by accessibility. Consequently, remote sensing has been an asset to the mapping of reedbed for several decades^18^. Reedbeds have typically been mapped at the scale of individual wetlands, typically achieving greatest accuracy by the use of LiDAR or optical imagery from unmanned aerial vehicles^19,20^. However, few remote sensing studies have mapped reedbed at larger scales; for example, one study mapped reedbed at a national scale in Hungary, but within known fishponds alone^21^. To our knowledge, no study has mapped this wetland type at the national scale across all habitat types.

The only estimate of the extent of reedbed in Britain (6,524 ha) stems from a field inventory of British reedbeds made in 1993^22,23^. The Priority Habitats’ Inventory (England)^17^ added to this inventory, within England alone, from a range of other local and regional recording schemes. However, these inventories focus solely on recording already known reedbeds and so are not exhaustive; little attempt was made to seek out unknown reedbeds. Here, we aim to map the extent of reedbed in Britain completely with a ‘top-down’ approach^4^, using remotely-sensed data. In doing this, we aim to provide a method that can be easily implemented and repeated by ecologists in the future, in order to estimate the change in distribution of this important land cover over time.

## Methods

### Study area, scale and data

We aimed to map all reedbed in Britain, at 10 m × 10 m scale (hereafter ‘10 m scale’). We acquired Level-1C imagery from the Multi-Spectral Instrument (MSI) of the Sentinel-2A satellite (at the time of analysis, the Sentinel-2B/2C sensors had not yet been integrated). Images for all 100 km × 100 km tiles overlapping any of the land surface of Britain and offshore islands except Rockall, captured during the period 1st October 2015–30th April 2017, with a given cloud cover of ≤ 5%, were downloaded. The long study period was selected to provide enough non-cloud coverage, over multiple seasons, for the whole study area. GDEM digital elevation model (DEM) data from the ASTER satellite (1 arc-second resolution; ~30 × 30 m at 55°N) were also downloaded for the study area.

### Pre-processing

Atmospheric correction of satellite data was image-based, and achieved by means of dark-object subtraction, implemented in QGIS^24^ using the Semi-Automatic Classification Plugin^25^. More advanced data processing options were not used, in order to implement a workflow that is accessible to those not expert in the field of remote sensing. Reed does not occur subtidally in Britain, and so all tiles were masked to land above the low tide mark.

To identify cloud, a random forest (500 trees, three variables tried at each split) was trained (using all 13 bands) on known cloud/non-cloud for one pass of one scene (30UYD on the military grid)^26^. Training areas of cloud and non-cloud for this pass were identified visually. This model had an out-of-bag error rate estimate of 1.1%. Cloud presence/absence was then predicted using this model across all scenes, and all predicted cloud pixels were masked out of all images.

Multi-temporal Sentinel-2A images can be mis-registered with respect to each other by up to three pixels at 10 m scale^27^, and need co-registering in order to compare by-pixel reflectances over time. Images were co-registered using the *coregisterImages* function in the R package *RStoolbox 0.1.10*^28^. Although the mis-registration was typically eliminated, for some images the function only marginally reduced the mis-registration, or made no improvement at all. Thus it is probably inevitable that our reflectances are slightly spatially smoothed when summarised over time, and our map probably misses some true narrow reedbeds. The co-registration function often failed when the non-NA content of the secondary rasters was below 3%, and especially below 1% (e.g. if the tile was mostly sea or cloud). Thus, four scenes for which the non-NA content of the secondary tile never reached above 3% were removed from analysis.

### Multi-temporal images

At any given time of the year, reedbed has broadly similar reflectance to other vegetation^29^. Much of the UK land area is dedicated to cultivation of grasses *Poaceae* for arable and animal agriculture (19% and 52% respectively in 2019^30^); these land cover types might have similar reflectance profiles to reedbed. However, reedbed’s reflectance and vegetation indices change distinctively through the seasons^31^, so by using data from more than one season classification uncertainty can be reduced^32^.

Ideally, we would have estimated reedbed’s temporal reflectance function for each band, and used that to predict reedbed presence/absence; however, two issues prevented this. Firstly, our cloud classification model could not identify cloud shadow, and so cloud shadows were removed by taking the median reflectance of several cloud-free images within seasons (thereby removing most temporal variation in reflectance from the dataset). Secondly, our study was carried out over 11 degrees of latitude; the temporal function of reedbed varies over relatively short distances^20,31,33^. Thus any true reedbed reflectance function is likely to vary across our study area, creating challenges for its estimation.

We split the dataset into two ‘seasons’; too few cloud-free images were available for some scenes to allow a higher temporal resolution. These seasons were designed to capture periods when reedbed is generally ‘green’ (‘summer’, June – September) or ‘brown’ (‘winter’, November – April) across the study area. Reedbed’s ‘green’ season is shorter in the north of the study area^33^; and so we left a month between the seasons, to avoid periods when reedbed might not be universally ‘green’ or ‘brown’ across the study area. The median reflectance was taken for each pixel of each scene for each season respectively.

### Training, classification and prediction

**Fig. 1:**
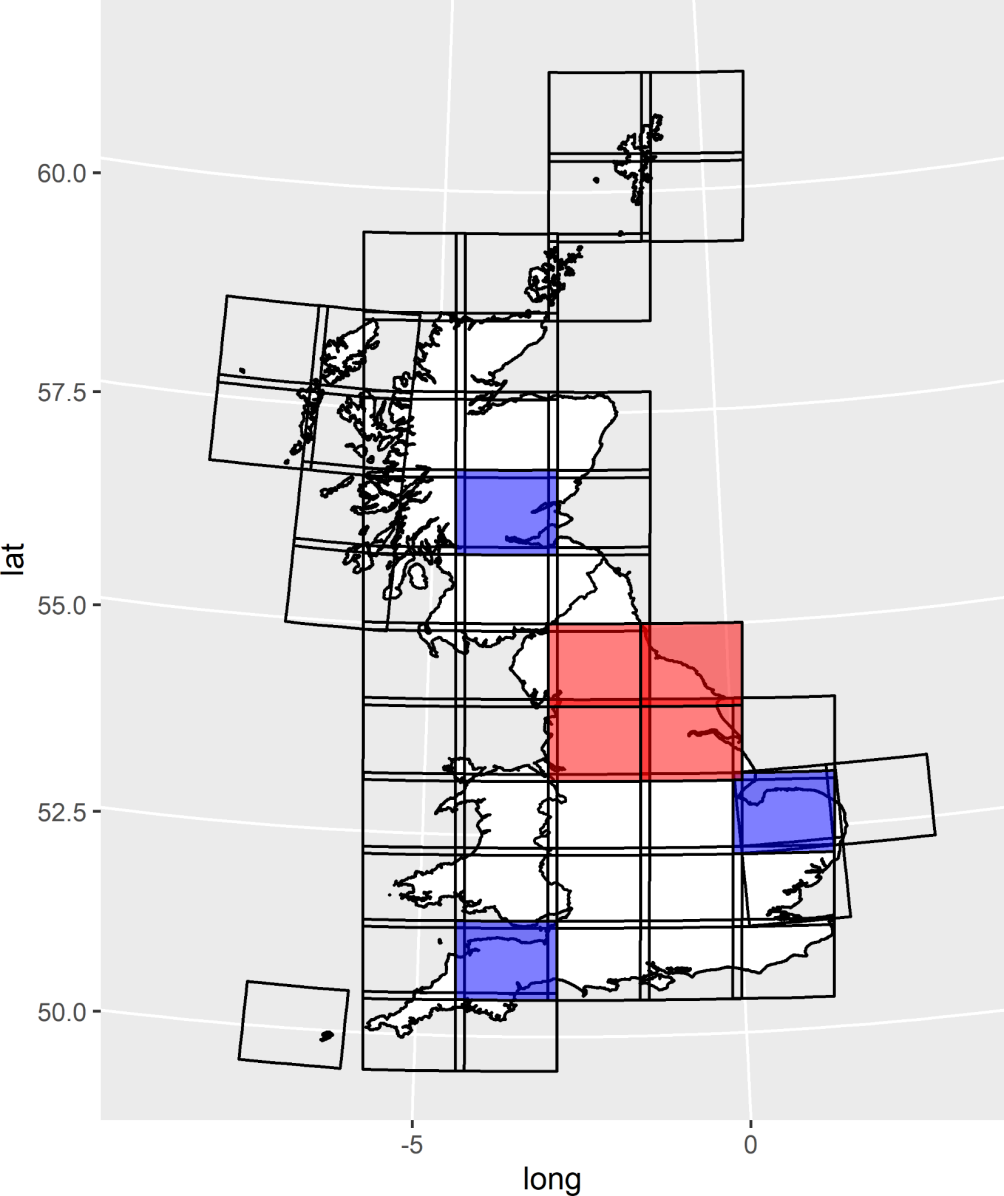
Map of scenes contributing to combined dataset, training scenes (blue) and validation scenes (red). Map created using R version 4.2.1^38^ (https://www.r-project.org/); coastline map from GADM (https://gadm.org/license.html).

173 training polygons (29 reedbed, 144 non-reedbed) with a total area of 2,798.4 ha (79.2 ha reedbed, 2719.2 ha non-reedbed) were identified from visual interpretation of Google Maps and Google Street View imagery (Google imagery 0–7 years old at the time) and from the authors’ personal knowledge. Non-reedbed polygons were selected from a range of different land cover types. Training polygons were added iteratively, until the predicted reedbed map within the training scenes changed little with each new polygon. These polygons were located on three different scenes (Fig. 1), selected so that they covered a wide geographical span (to allow for geographical variation in reedbed’s reflectance and phenology) and variety of non-reedbed land cover types, and so that they contained a reasonable extent of reedbed. Edges of training polygons were located away from reedbed/non-reedbed boundaries to avoid co-registration errors. Training the classification model using free online imagery avoids one of the two field data collection campaigns associated with remote sensing land cover classification, and the associated financial and time costs.

The 1993 reedbed inventory^22^ and subsequent update in England^17^ are not exhaustive (e.g. omitting some English reedbeds known to the authors), and some reedbeds therein may not exist anymore. Records of Reed held by the Botanical Society of Britain and Ireland consist of point locations, but contiguous polygons of reedbed are needed for training data. Therefore we considered that online imagery provided a more appropriate dataset than these inventories for training and validation.

A random forest (500 trees, three variables tried at each split) was trained on known reedbed/non-reedbed. Random forest was chosen over other classification algorithms (such as deep learning) to maximise ease of implementing our workflow by a wide range of users. The model was trained at points sampled at random from within the polygons: 100 points from each polygon; points in the same pixel as another point were then discarded (median points remaining per polygon: reedbed, 71; non-reedbed, 91). Using band ratios or indices, rather than raw bands, can avoid noise from natural absolute variation in irradiance over multiple dates^34^, potentially improving classification methods. No single index has proved useful in mapping reedbed across previous studies^31,35^, so our combined dataset consisted of seven indices, band ratios and standardised bands (Supplementary Table S1) for each season, and the difference in SAVI and NDWI between seasons.

Two studies^32,36^ found that incorporating texture information improved reedbed classification accuracy. However, these studies used much finer resolution data (2.4 m × 2.4 m pixels) to map large reedbeds. We sought to map reedbeds down to the size of one Sentinel 2-A pixel (10 m × 10 m) – sometimes the full extent of a reedbed. Classification using texture information could therefore only have been performed for a subset of larger reedbeds, which was not the aim of our study, and we did not pursue this approach.

Using the trained classification model, reedbed presence probability was predicted across Britain for each scene using the combined dataset. The probability threshold which maximised Cohen’s kappa was calculated for the model: predicted reedbed presence (*p*) was ‘0’ or ‘1’ if respectively above or below this threshold. Iterative training and re-prediction to improve visual accuracy was carried out until the model could not be improved. As reedbed polygons were added, the rate of decline in the ‘reedbed’ out-of-bag commission error rate slowed while the rate of increase in the ‘non-reedbed’ commission error rate stayed the same (Figure S1). This suggests that although we only used 29 training polygons for reedbed, adding more reedbed polygons to the training data would not have improved overall accuracy.

After validation (see below), predicted reedbed maps were aggregated to hectare (100 m × 100 m) scale, before being re-projected to WGS 84 UTM zone 30 and mosaicked together. Ground slope was calculated from the digital elevation model (DEM), and the slope map and DEM were re-projected to the same datum and scale as the predicted reed map. Any cells with a slope of more than 10° or an altitude of more than 470 m (maximum recorded altitude of Reed in Britain^7^) were assigned *p* = 0.

### Workflow in Google Earth Engine

Google Earth Engine (‘GEE’^37^) has become widely used in recent years. GEE uses cloud services to massively scale up computational capability for geospatial analysis, presenting two key advantages over our workflow carried out on a local machine and high-performance computing cluster (hereafter ‘HPC workflow’): much greater data storage capacity and much greater processing speed.

The proportion of Sentinel-2 data that could be incorporated in our HPC workflow was limited by storage capacity. Therefore certain scenes only had a small number of cloud-free passes for a given season, and so the temporal resolution of the data on which the random forest could be trained was limited. We therefore repeated the entire HPC workflow with GEE: to attempt to generate a more accurate reedbed map, and to assess the extent to which relaxing data constraints improves the accuracy of geospatial analysis.

In the GEE workflow we used all Sentinel-2 data (S-2A & B), from the initiation of the Sentinel-2 program (28th June 2015) until the date of analysis (27th July 2019). Temporal resolution was increased from two periods to four: February-March; June-July; August-September; November-December. The combined dataset comprised NDWI, EVI, SAVI, RG, GB, NDVI and SB4 (inter-seasonal differences in SAVI and NDWI were not used, because there were more seasons). GEE imposes user memory limits for tasks, so there was a trade-off between: maximising the number of variables; further increasing the number of images (by increasing the maximum acceptable cloud cover to ≤ 25%); or further increasing the temporal resolution (to eight periods: February, March, June, July, August, September, November and December). One classification model was run for each of these data maximisation approaches, and the accuracy of the resulting map assessed (see validation process below).

The GEE workflow was kept as similar as possible to the HPC workflow. However, some aspects were unavoidably different. Pixels were sampled within polygons (rather than points within polygons), and so it was not possible to balance sampling between categories. It was not straightforward to buffer a raster land mask in GEE, so we used the British shoreline rather than the shoreline plus 100 m buffer: therefore some coastal reedbeds may be missed. The GEE facility to predict probabilities with random forest was not available at the time of analysis, and so presence/absence was predicted. Steep/high altitude terrain was removed from the data stack before training and prediction. We used GEE’s cloud and cirrus removal tools. Because we varied the number of variables between the three data maximisation approaches, we set the random forest to the default setting of number of variables per split: the square root of the number of variables.

### Validation

Covering 11 degrees of latitude (~ 1,200 km), it was unfeasible with available resources to validate the map across the entire study area; instead the map was validated across four scenes in northern England (Fig. 1). The validation area was selected according to the following criteria: to maximise geographical distance from the training polygons, for independence therefrom; to contain a wide range of the habitats present in Britain while being central within the study area and spanning > 100 km between the most distant validation locations, so validation statistics would be as representative as possible; to cover several scenes, to account for variation in validation statistics between scenes due to reflectance error.

Reedbed is a rare land cover type nationally, and thus sampling a random selection of pixels would find too few cells with non-zero probability of reedbed occurrence to estimate either commission error or omission error accurately. Fieldwork was therefore targeted disproportionately towards cells with non-zero probability of reedbed presence. To estimate commission error, 40 hectares were selected from each scene, with 10 randomly selected from within each of the following ranges of predicted proportion (*p*) of reedbed cover from the predicted map: *p* = 0; 0 < *p* ≤ 0.33; 0.33 < *p* ≤ 0.66; 0.66 < *p* ≤ 1. To minimise travel costs, these were selected from the quarter of the scene with the highest non-zero probability of reedbed occurrence.

Each of these hectares was visited and both reedbed (total area of contiguous Reed) and Reed cover (total area of any Reed patches) of the hectare were visually estimated to the nearest 10%. Then, in each hectare, six randomly selected 10 m cells (three predicted reedbed and three predicted non-reedbed) were visited and reedbed (defined as ≥ 1 m^2^ contiguous Reed) and Reed presence were recorded (in practice these were the same). Commission error was quantified in two ways. At the ha scale, predicted and observed reedbed cover were regressed, and the coefficient of determination (R^2^) was computed. At the 10 m scale, predicted and observed presences were compared, to give an area under the curve (AUC) of the receiver operating characteristic for reedbed cover. Validation fieldwork was carried out from 6th October 2017 to 2nd November 2017.

For the HPC workflow, visual inspection of candidate random forests showed that balanced and unbalanced random forests, and random forests with slightly different training areas, had similar areas of true positives and slightly different areas of false positives. Thus, in order to reduce the area of false positives, two different random forests (‘RF1’ and ‘RF2’) were used for the final model, and the final map was created using the minimum predicted reedbed probability of the two random forests, for each pixel.

The validation fieldwork had already taken place before the GEE workflow was carried out. The validation data were used to assess the accuracy of the GEE reedbed map. The GEE reedbed maps and the validation data are both at 10 m × 10 m resolution, but they have slightly different origins and projections. To assess accuracy at the ha scale, the GEE reedbed map was projected onto the validation map, and predicted reedbed cover was regressed against observed reedbed cover. To assess accuracy at the 10 m scale, the centre points of the 10 m validation cells were re-projected onto the GEE reedbed map, and the predicted and observed presences were compared. As presence/absence (rather than probability) was predicted, AUC could not be calculated for the GEE reedbed map.

All data availability queries, download, classification, raster manipulation and random selection were carried out in R 4.2.1^38^; pre-processing was carried out in R and QGIS v2.18.

## Results

### HPC workflow—structure of random forests

**Fig. 2:**
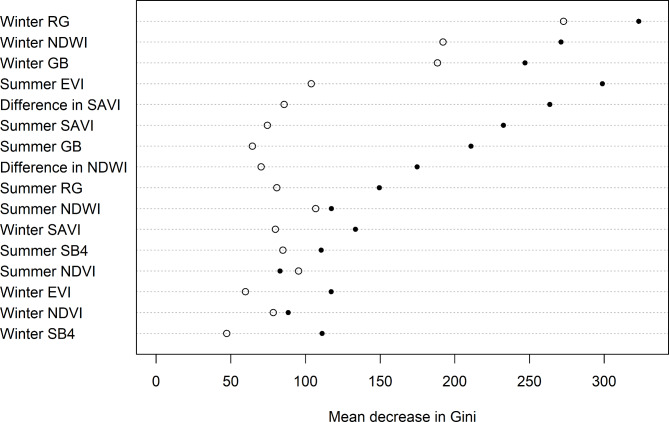
Importance of variables in the two final random forests contributing to the final map (filled circles = RF1; open circles = RF2), HPC workflow. A higher decrease in Gini denotes a greater variable importance.

Winter reedbed spectral characteristics (red-green ratio, RG; normalised difference water index, NDWI; green-blue ratio, GB) constituted two or three of the most informative variables in RF1 and RF2 respectively (Fig. 2). In RF2 the three most informative variables were considerably more informative than the others, but in RF1 there was less separation between these and the remainder. Kappa was maximised at a probability of 0.585 – this was used as the threshold for classifying 10 m pixels.

### Accuracy of HPC reedbed map

Files from 541 passes were acceptable for use after pre-processing (mean 10.82 passes/scene, range 3–22). Each scene had at least one winter and one summer pass. The random forests were predicted over indices derived from these data.

**Fig. 3:**
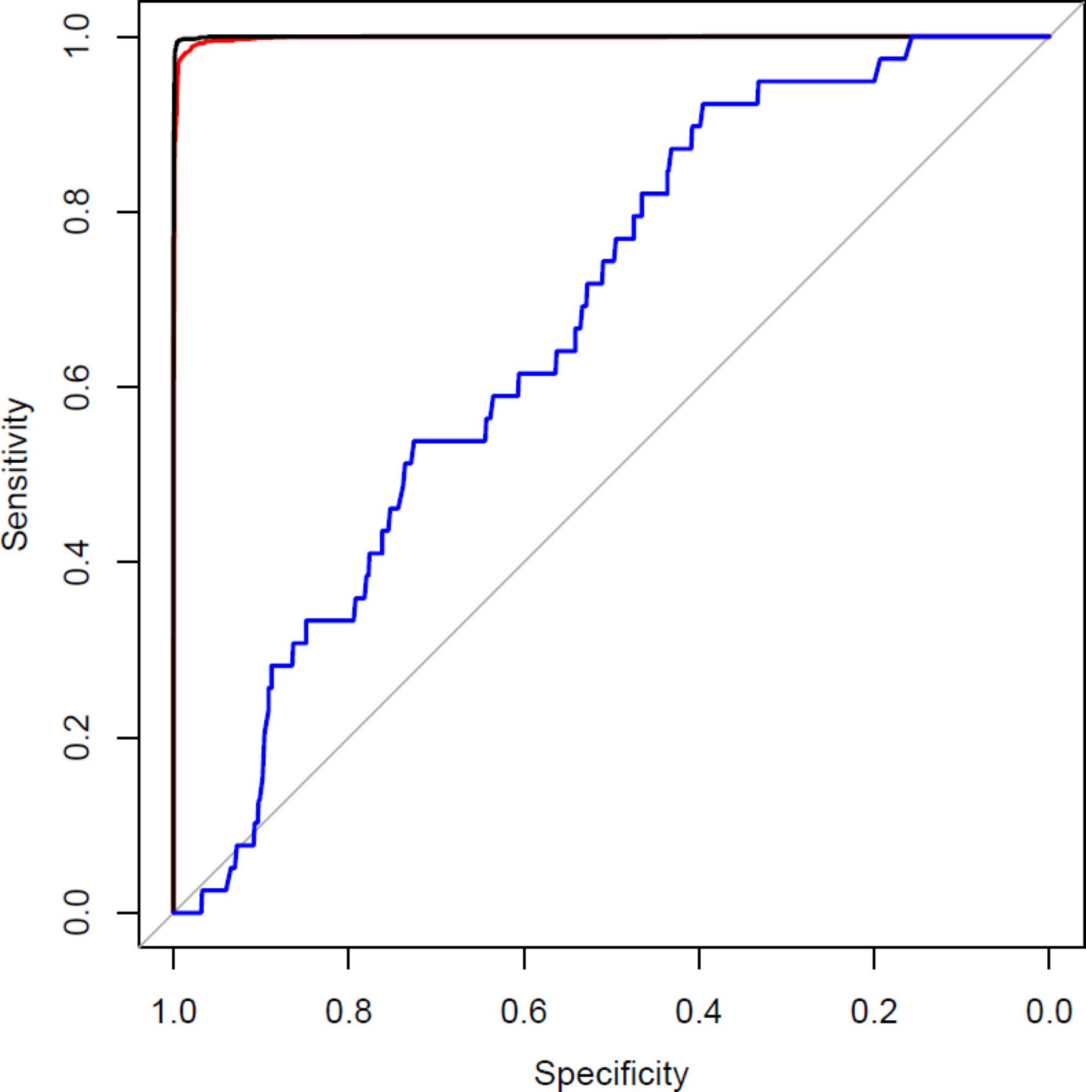
Receiver operating characteristic curve for 10 m pixels, HPC workflow: black, map against RF1 training data; red, map against RF2 training data; blue, map against validation data. Sensitivity = proportion of true positives that are correctly classified; specificity = proportion of true negatives that are correctly classified.

When tested against the training data, the classification model had near-perfect discrimination: the two random forests used respectively had AUC values of 0.9997 (RF1) and 0.9983 (RF2) against the final map (Fig. 3). Only 154 validation hectares were visited in total, for two reasons: for one validation scene, only eight hectares had a predicted probability of 0.66 < *p* ≤ 1; two validation hectares from other scenes were not safely accessible. When tested against the validation data, the classification model had much lower discrimination: the combined model had an AUC of 0.671 (Fig. 3, blue line). The overall accuracy of the map at the 10 m scale was 65.1%, but the commission error for reedbed was very high: the majority of predicted reedbed was not true reedbed (Table 1a).

The commission error for reedbed remained very high at the ha scale (Table 1b), although slightly lower than at the 10 m scale. The class frequency of predicted reedbed is deliberately over-represented in our sample (see Materials and Methods), and so overall accuracy and omission error are not presented for the ha scale map, because they would be respectively over- and under-estimated. The estimated proportion of reedbed in the landscape was much greater (0.344 at 10 m scale; 0.740 at ha scale) than the true proportion (0.042 at 10 m scale; 0.090 at ha scale).

**Table 1:** Confusion matrix for reedbed map at: (a) 10 m scale; (b) ha scale (proportional reedbed cover converted to binary presence/absence).

a) 10 m scale							
		Predicted					
		Reedbed		Not reedbed		Omission error (%)	
		HPC	GEE	HPC	GEE	HPC	GEE
**Observed**	Reedbed	21	10	18	29	46.1	74.4
	Not reedbed	297	17	588	868	33.5	1.9
	Commission error (%)	93.4	63.0	3.0	3.2		

b) Hectare scale							
		Predicted					
		Reedbed		Not reedbed			
		HPC	GEE	HPC	GEE		
**Observed**	Reedbed	11	6	2	7		
	Not reedbed	103	10	38	131		
	Commission error (%)	90.3	62.5	5.0	5.1		

**Fig. 4:**
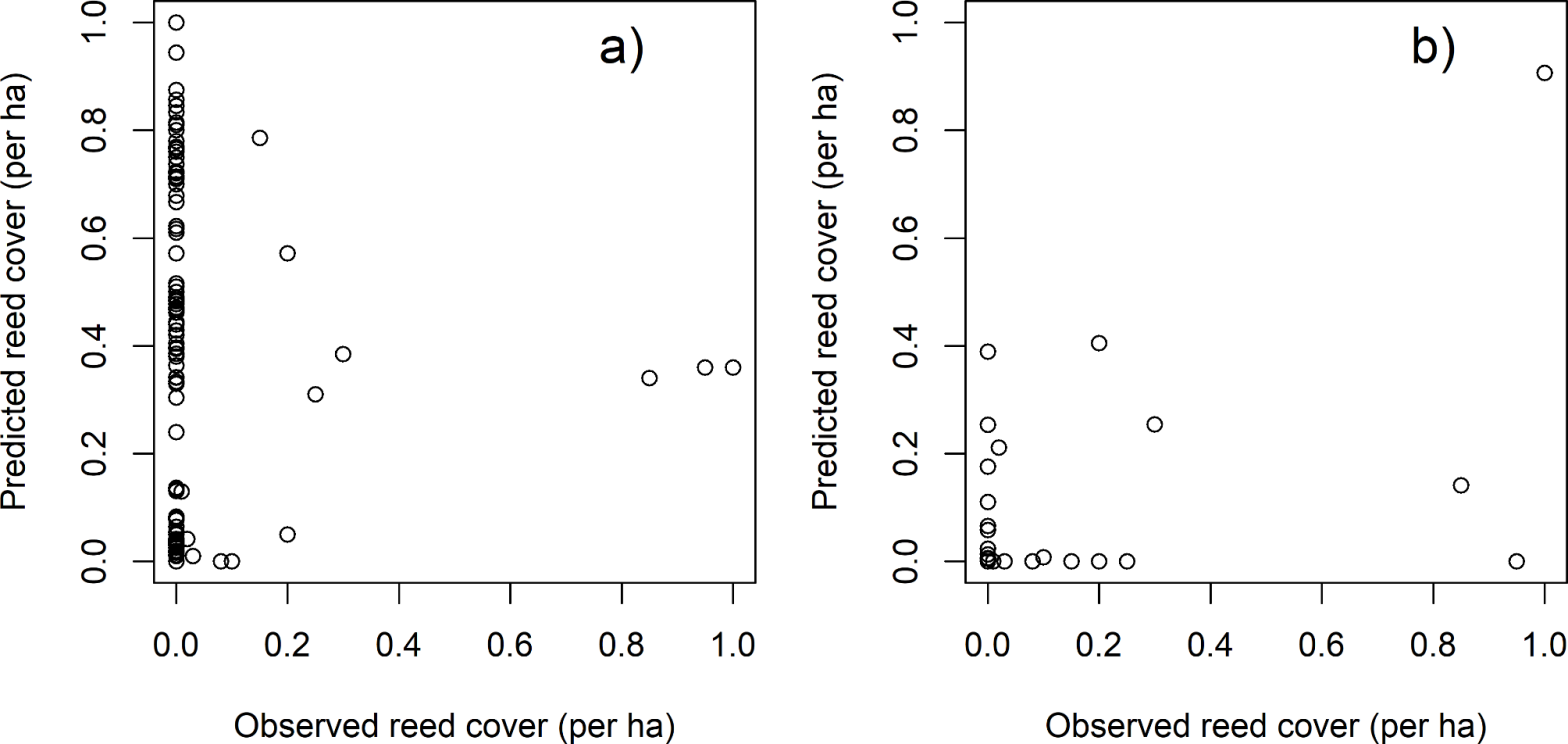
Predicted and observed ha scale reedbed cover: (**a**) HPC workflow, (**b**) GEE workflow.

There was no relationship between predicted and observed reedbed cover at the ha scale (Fig. 4a). False positives were non-randomly spread among land cover types (Chi-square test, Χ^2^ (1, *N* = 8) = 32.0, *p* < 0.0001; Supplementary Table S2). Arable and other open land cover types comprised 61.7% of the sample squares but 73.8% of the false positives.

### HPC workflow—distribution and extent of reedbed

The total area predicted to be covered by reedbed, including known error, is 54,273 ha. Assuming 93.4% commission error and 46.1% omission error, we estimate that 7,765 ha of Britain is covered by reedbed.

**Fig. 5:**
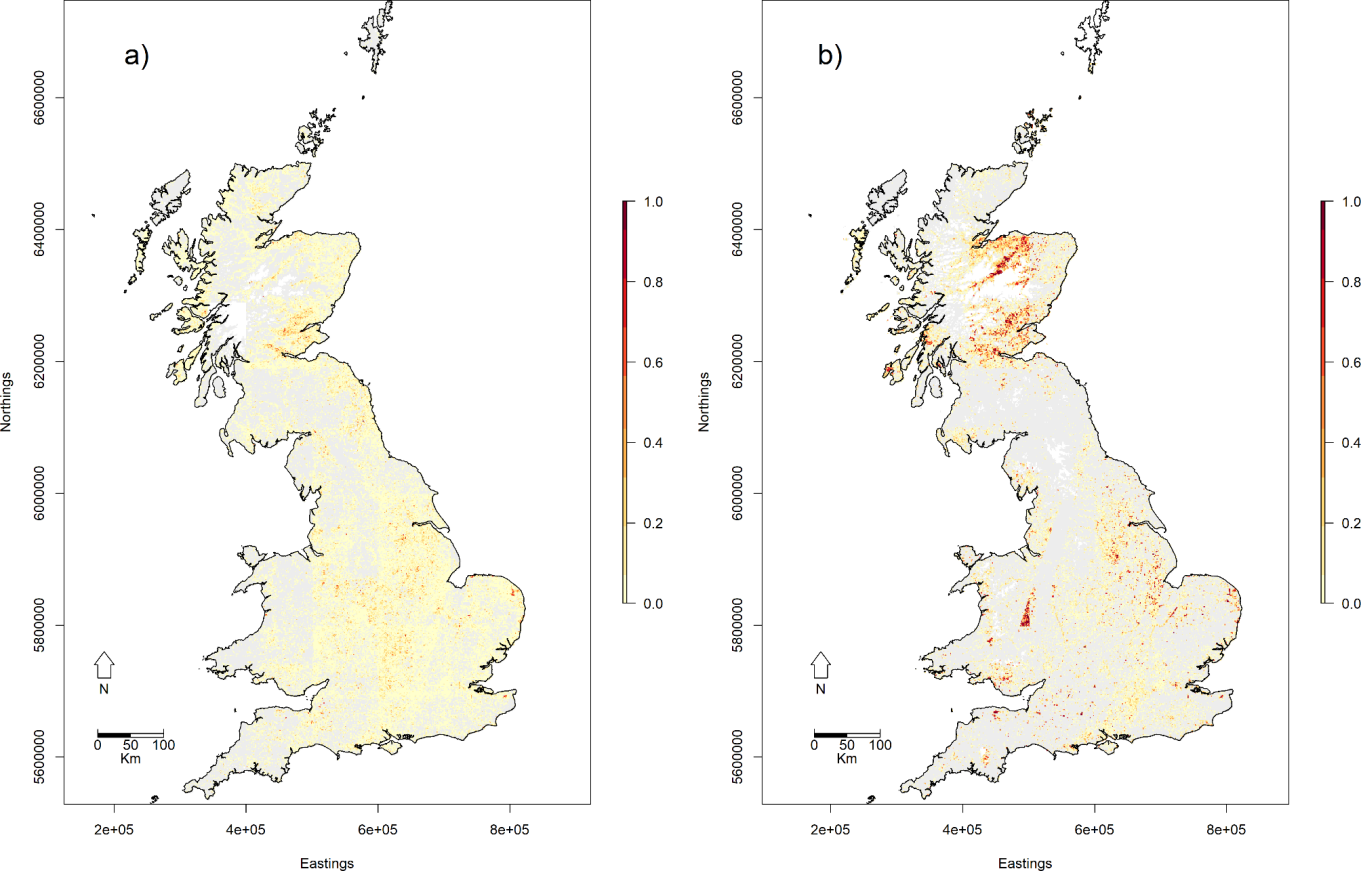
Predicted reedbed map (colour = maximum predicted per-ha cover at 1 km^2^ scale; grey = 0; white = NA) of Britain: (**a**) HPC workflow; (**b**) GEE workflow. Steep/high altitude terrain has been masked out. Coastline map from GADM (https://gadm.org/license.html). Contains modified Copernicus Sentinel data (2015–2019). Sentinel-2 data were processed using R version 4.2.1^38^ (https://www.r-project.org/), QGIS version 2.18^24,25^ (https://qgis.org/), and Google Earth Engine^37^ (https://earthengine.google.com/).

**Fig. 6:**
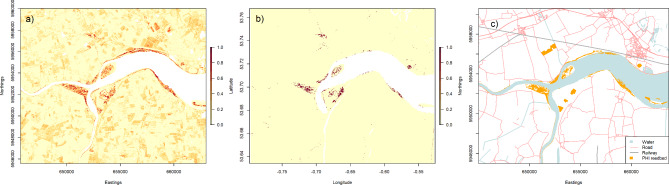
Predicted and documented reedbed in the upper Humber estuary, NE England: predicted reedbed map (colour = predicted probability of reedbed presence at 10 m scale; white = NA) for (**a**) HPC workflow and (**b**) GEE workflow; (**c**) PHI reedbed polygons^17^ (base map data from OpenStreetMap https://www.openstreetmap.org/copyright). Contains modified Copernicus Sentinel data (2015–2019). Sentinel-2 data were processed using R version 4.2.1^38^ (https://www.r-project.org/), QGIS version 2.18^24,25^ (https://qgis.org/), and Google Earth Engine^37^ (https://earthengine.google.com/).

Subsequent masking of sloping or high-altitude cells removed a median of 50.8% (range 9.5 − 87.2%) of predicted reedbed for each scene. The distribution of reedbed in Britain estimated using the HPC workflow is presented in Fig. 5a. An example subset of the map at the Humber estuary, England, is presented in Fig. 6a (PHI reedbed polygons for comparison in Fig. 6c).

### GEE workflow

It was not possible to balance sampling between categories in the GEE workflow. Therefore only one, unbalanced, random forest was run for each set of criteria. Of the three data-maximisation approaches carried out (maximising number of variables, number of images or temporal resolution), maximising the number of variables gave both the lowest commission error and the lowest omission error: this approach was used. 5,184 images were used, 9.6 times as many as used in the HPC workflow.

The distribution of reedbed in Britain estimated using the GEE workflow is presented in Fig. 5b. An example subset of the GEE reedbed map at the Humber estuary, England, is presented in Fig. 6b (PHI reedbed polygons for comparison in Fig. 6c). At the 10 m scale, the best reedbed map produced by the GEE workflow had an overall accuracy of 93.8%. This is higher than the overall accuracy of the HPC workflow 10 m scale reedbed map, but is almost identical to the overall accuracy of a map classifying all the validation points as non-reedbed (94.1%). At the 10 m scale the estimated proportion of reedbed (0.029) in the landscape was lower than the true proportion (0.042), but at the ha scale the estimated and true proportions of reedbed were very similar (0.104 and 0.090 respectively). The commission error (63.0%) was still high (Table 1a), but considerably lower than that of the HPC workflow reedbed map. However, the GEE omission error was higher than that of the HPC workflow reedbed map: nearly three-quarters of observed reedbed was predicted not to be reedbed (Table 1a). The increase in omission error relative to that of the HPC workflow map is proportionately larger than the reduction in commission error.

At the ha scale, the best reedbed map produced by the GEE workflow had a similar accuracy to the 10 m scale map (Table 1b, Fig. 5b). There was a weak positive correlation (r = + 0.57) between predicted and observed reedbed cover (Fig. 4b), although this is again likely to be due to the largely correct identification of non-reedbed, and the high true class frequency of non-reedbed.

## Discussion

Remote sensing studies refined to individual wetlands or groups of wetlands have typically had very high accuracy in identifying reedbed (e.g. 92%^21^, 94.7%^19^, 97%^29^, 98.7%^35^), potentially because of their small geographical scale. By contrast, field validation revealed high commission and omission error in our reedbed map. Surprisingly, repeating our workflow in Google Earth Engine using almost an order of magnitude more data from both Sentinel-2 sensors (only data from S-2A were available for the original workflow), and using more sophisticated pre-processing methods, did not resolve the accuracy issues. Although the quantity disagreement was reduced in the GEE workflow map, the allocation disagreement was increased. We argue that this lower accuracy in our map stems from the relatively large geographical area covered by our study, resulting in a greater number of confusion land cover types and greater spatial variation in temporal reflectance function, and encompassing a geographical scale at which absolute reflectance error between satellite swaths and scenes is relevant.

Our study area, spanning 11 degrees of latitude, covers a wide range of semi-natural and man-made land cover types including grasses other than reed. These constitute a much larger set of land cover types with similar reflectance profiles to reedbed (as evidenced in the high commission error in these land cover types) than is present in a single wetland, making reedbed relatively less distinctive and elevating commission error accordingly. Similarly, reedbed’s relative rarity means that even with a low commission error rate the total area of incorrect commissions would dwarf the total area of correct commissions. This problem is likely to apply generally when classifying other rare and localised land cover types, which are often of disproportionate conservation value.

Reed can vary dramatically in phenology over very small spatial scales^20^, and there is spatial variation in reedbed’s temporal reflectance function across our study area^7^. This potentially makes reedbed’s temporal reflectance function so variable as to overlap with that of other land cover types, preventing a classification algorithm from discriminating between them. This seems to be a key barrier to correctly classifying reedbed at large geographical scales, because increasing the temporal resolution of the data (using GEE) did not improve the accuracy of the map. This issue may also apply generally to vegetation types whose temporal reflectance functions are easily distinguished from those of other vegetation cover over small spatial areas, but which are variable over large geographical areas. This issue could theoretically be resolved by additionally incorporating hyperspectral or non-optical data, or by using texture information if the aim was classification of large (relative to the pixel size) reedbeds alone. However, including SAR data from the Sentinel-1 satellite in preliminary analyses did not improve the GEE map, and no freely available hyperspectral or LiDAR products exist for the whole of Britain. Future analyses could include latitude as a covariate in a temporal reflectance function.

Reflectance error (e.g. due to instrument error, variation in solar radiation, or cloud removal error) compromises geospatial analysis when the study area is large enough to include multiple scenes. This was evidenced in our study by the visible presence of swath and scene boundaries in the reedbed map (Fig. 5a). This was not resolved by massively increasing the number of passes in the dataset with GEE, in order to bring the estimated median reflectance or reflectance-derived measure closer to the true median (Fig. 5b). Reflectance error therefore limits the possibility for predicting outside the swath or scene in which a classification model has been trained.

Beyond a certain training dataset size, adding more training data typically has diminishing positive effects on classifier performance^39^. In our study, the classification error issues could not be resolved by adding more reedbed training polygons: doing so had a diminishing negative effect on reedbed commission error rate, which did not compensate for increases in non-reedbed commission error rate. It could be that adding new reedbed training data introduced noise in the form of ecological and observation-error-induced variation in reedbed’s reflectance. This could then have limited the model’s ability to improve at identifying reedbed with new data, while simultaneously incorrectly suggesting new apparent ‘reedbed’ reflectance patterns in truly non-reedbed areas. This trade-off is likely to be a challenge when choosing training dataset size for the classification of other geographically-dispersed rare habitat types.

Our reedbed map, which had better-than-random accuracy (as assessed using AUC) at predicting the presence of a wetland land cover type, hundreds of kilometres from the training areas, is the first of Britain and the first to our knowledge at such a large spatial scale. However, due to the high classification error, it is more appropriate to use our map (in conjunction with estimated commission and omission error) for a first overall estimate of the total extent of reedbed in Britain, rather than for describing the location of individual reedbeds. Our estimate (7,765 ha) is the first comprehensive estimate of reedbed extent in Britain, and is of a similar order of magnitude (6,524 ha) to the only other estimate of reedbed extent in Britain (from 1993)^22^. It is not clear whether the 19.0% increase between the two estimates is due to: a real increase in reedbed in Britain since 1993; the limitation of the previous estimate^22^ to known reedbeds; or error in our classification process.

The utility of our map for identifying the fine-scale location of reedbed could be improved by masking confusion land cover types such as arable out with agricultural maps. Repeating our analysis in the future could allow assessment of national change in reedbed extent for natural capital accounting. However, although we sought to make our validation statistics as representative as possible by our selection of the validation area, our estimate of the extent of reedbed in Britain (and thus future estimates calculated by the same method) is highly contingent on our estimates of commission and omission error and must therefore be used with caution.

Low classification accuracy continues to limit the utility of some large geographical scale land cover maps^40^. Although repeating our workflow in Google Earth Engine did not resolve such classification accuracy issues, it brought the total analysis time down from several weeks to less than a day, and eliminated the need for large data storage capacity. Classification accuracy for a workflow like ours may improve considerably in the future as satellite reflectance error is reduced, and may improve slightly if other classifiers such as deep learning are used rather than random forest^41^. However, some sources of error remain which are clearly not easily resolved by advances in data quality or analytical techniques: ecological factors such as the number of confusion land cover types and systematic variation in temporal vegetation reflectance functions probably place upper limits on the size of a geographical area that can be classified accurately with such a workflow. Ultimately, this may fundamentally limit the capacity for remote sensing to aid and inform ecological resource management.

## Supplementary Information

Supplementary Material 1

## Data Availability

This study used Sentinel-2 data which are publicly available at the Copernicus Open Access Hub (scihub.copernicus.com) or through Google Earth Engine (earthengine.google.com). Field data used in validation are available at https://github.com/btojacobdavies/GB_reedbed_map.

## References

[1] Fluet-Chouinard, E. et al. Extensive global wetland loss over the past three centuries. *Nature*. **614**, 281–286 (2023). 36755174 10.1038/s41586-022-05572-6

[2] Clarkson, B. R., Ausseil, A. G. E. & Gerbeaux, P. Wetland ecosystem services. in Ecosystem Services in New Zealand—conditions and Trends (ed Dymond, J. R.) 192–202 (Manaaki Whenua, Lincoln, New Zealand, (2013).

[3] Davidson, N. C. How much wetland has the world lost? Long-term and recent trends in global wetland area. *Mar. Freshw. Res.* **65**, 934–941 (2014).

[4] Davidson, N. C., Fluet-Chouinard, E. & Finlayson, C. M. Global extent and distribution of wetland: Trends and issues. *Mar. Freshw. Res.* **69**, 620–627 (2018).

[5] Convention on Biological Diversity. First Draft of the Post-2020 Global Biodiversity Framework (2021).

[6] Hu, S., Niu, Z. & Chen, Y. Global wetland datasets: A review. *Wetlands*. **37**, 807–817 (2017).

[7] Packer, J. G., Meyerson, L. A., Skálová, H., Pyšek, P. & Kueffer, C. Biological Flora of the British Isles: *Phragmites australis*. *J. Ecol.* **105**, 1123–1162 (2017).

[8] Brix, H., Sorrell, B. K. & Lorenzen, B. Are phragmites-dominated wetlands a net source or net sink of greenhouse gases? *Aquat. Bot.* **69**, 313–324 (2001).

[9] Köbbing, J. F., Thevs, N. & Zerbe, S. The utilisation of reed (*Phragmites australis*): A review. *Mires Peat*. **13**, 1–14 (2013).

[10] Chambers, R. M., Meyerson, L. A. & Saltonstall, K. Expansion of *Phragmites australis* into tidal wetlands of North America. *Aquat. Bot.* **64**, 261–273 (1999).

[11] Meyerson, L. A., Cronin, J. T. & Pyšek, P. *Phragmites australis* as a model organism for studying plant invasions. *Biol. Invasions*. **18**, 2421–2431 (2016).

[12] Martin, L. J. & Blossey, B. The runaway weed: Costs and failures of *Phragmites australis* management in the USA. *Estuaries Coasts*. **36**, 626–632 (2013).

[13] Lansdown, R. V. *Phragmites australis*. In *The IUCN Red List of Threatened Species* (2017). 10.2305/IUCN.UK.2017-3.RLTS.T164494A121712286.en (2017).

[14] Van Der Putten, W. H. Die-back of *Phragmites australis* in European wetlands: An overview of the European Research Programme on Reed die-back and progression (1993–1994). *Aquat. Bot.* **59**, 263–275 (1997).

[15] Vermaat, J. E., Bos, B. & Van Der Burg, P. Why do reed beds decline and fail to re-establish? A case study of Dutch peat lakes. *Freshw. Biol.* **61**, 1580–1589 (2016).

[16] Morganti, M. et al. Multi-species habitat models highlight the key importance of flooded reedbeds for inland wetland birds: implications for management and conservation. *Avian Res.* **10**, 15 (2019).

[17] Natural England. Priority Habitats Inventory (England) (2023).

[18] Butera, M. K. Remote sensing of wetlands. *IEEE Trans. Geosci. Remote Sens.* **GE-21**, 383–392 (1983).

[19] Higgisson, W., Cobb, A., Tschierschke, A. & Dyer, F. Estimating the cover of *Phragmites australis* using unmanned aerial vehicles and neural networks in a semi-arid wetland. *River Res. Appl.* **37**, 1312–1322 (2021).

[20] Tóth, V. R. Monitoring spatial variability and temporal dynamics of phragmites using unmanned aerial vehicles. *Front. Plant. Sci.* **9**, 1–11 (2018). 29915608 10.3389/fpls.2018.00728 PMC5994432

[21] Sharma, P. et al. Estimating Reed Bed Cover in Hungarian fish ponds using NDVI-Based remote sensing technique. *Water*. **15**, 1554 (2023).

[22] Painter, M., Smith, K., & Gilbert, G. *An Inventory of British Reedbeds 1993* (1995).

[23] Bibby, C. J. & Lunn, J. Conservation of reed beds and their avifauna in England and Wales. *Biol. Conserv.* **23**, 167–186 (1982).

[24] QGIS Development Team. QGIS Geographic Information System (2017).

[25] Congedo, L. Semi-automatic classification plugin documentation (2016). 10.13140/RG.2.2.29474.02242/1

[26] Liaw, A. & Wiener, M. Classification and regression by randomForest. *R News*. **2**, 18–22 (2002).

[27] Skakun, S., Roger, J. C., Vermote, E. F., Masek, J. G. & Justice, C. O. Automatic sub-pixel co-registration of Landsat-8 operational land Imager and Sentinel-2A multi-spectral instrument images using phase correlation and machine learning based mapping. *Int. J. Digit. Earth* 1–17 (2017). 10.1080/17538947.2017.1304586 PMC6999662 32021650

[28] Leutner, B., Horning, N. RStoolbox: Tools for Remote Sensing Data Analysis (2017).

[29] Gilmore, M. S. et al. Integrating multi-temporal spectral and structural information to map wetland vegetation in a lower Connecticut River tidal marsh. *Remote Sens. Environ.* **112**, 4048–4060 (2008).

[30] DEFRA. *Agriculture in the United Kingdom 2019* (Department for Environment Food and Rural Affairs, 2020).

[31] Villa, P., Laini, A., Bresciani, M. & Bolpagni, R. A remote sensing approach to monitor the conservation status of lacustrine *Phragmites australis* beds. *Wetl. Ecol. Manag.*. **21**, 399–416 (2013).

[32] Onojeghuo, A. O., & Blackburn, G. A. Remote sensing of reedbeds. In *Remote Sensing for Science, Education, and Natural and Cultural Heritage: Proceedings of the 30th Symposium of European Association of Remote Sensing Laboratories* 38–43 (Paris, France, 2010).

[33] Haslam, S. M. Phragmites communis trin. *J. Ecol.* **60**, 585–610 (1972).

[34] Singh, A. Digital change detection techniques using remotely-sensed data. *Int. J. Remote Sens.* **10**, 989–1003 (1989).

[35] Davranche, A., Lefebvre, G. & Poulin, B. Wetland monitoring using classification trees and SPOT-5 seasonal time series. *Remote Sens. Environ.* **114**, 552–562 (2010).

[36] Onojeghuo, A. O. & Blackburn, G. A. Exploiting high resolution multi-seasonal textural measures and spectral information for reedbed mapping. *Environments*. **3**, 5 (2016).

[37] Gorelick, N. et al. Google Earth Engine: Planetary-scale geospatial analysis for everyone. *Remote Sens. Environ.* **202**, 18–27 (2016).

[38] R Core Team. *R: A Language and Environment for Statistical Computing* (R Foundation for Statistical Computing, 2024).

[39] Ramezan, C. A., Warner, T. A., Maxwell, A. E. & Price, B. S. Effects of training set size on supervised machine-learning land-cover classification of large-area high-resolution remotely sensed data. *Remote Sens.* **13**, 368 (2021).

[40] Liu, L. et al. Finer-resolution mapping of global land cover: Recent developments, consistency analysis, and prospects. *J. Remote Sens.* 1–38 (2021).

[41] Venter, Z. S., Barton, D. N., Chakraborty, T., Simensen, T., & Singh, G. Global 10 m land use land cover datasets: A comparison of dynamic world, world cover and esri land cover. *Remote Sens.* **14** (2022).

