# Supplementary material for: Estimating the distribution of reedbed in Britain demonstrates challenges of remotely sensing rare land cover types at large spatial scales
Source: Sci Rep. 2024 Sep 27;14:22271. doi: 10.1038/s41598-024-73030-6 (PMC11437167; doi:10.1038/s41598-024-73030-6)
Supplement: Supplementary file 1 — Supplementary Material 1 [file 41598_2024_73030_MOESM1_ESM.pdf]

Supporting Information

Table S1. Members of the combined dataset, and formulae used to derive them from individual bands (B).

Quantity	Formula
**Enhanced vegetation index (EVI)**	2.5*((B8-B4)/(B8+(6*B4)-(7.5*B2)+1))
**Green-blue ratio (GB)**	B3/B2
**Normalised difference vegetation index (NDVI)**	(B8-B4)/(B8+B4)
**Normalised difference water index (NDWI)**	(B3-B8)/(B3+B8)
**Red-green ratio (RG)**	B4/B3
**Soil-adjusted vegetation index (SAVI)**	(1.5*(B8-B4))/(B8+B4+0.5)
**Standardised blue band (SBB)**	B4/(B2+B3+B4+B8)

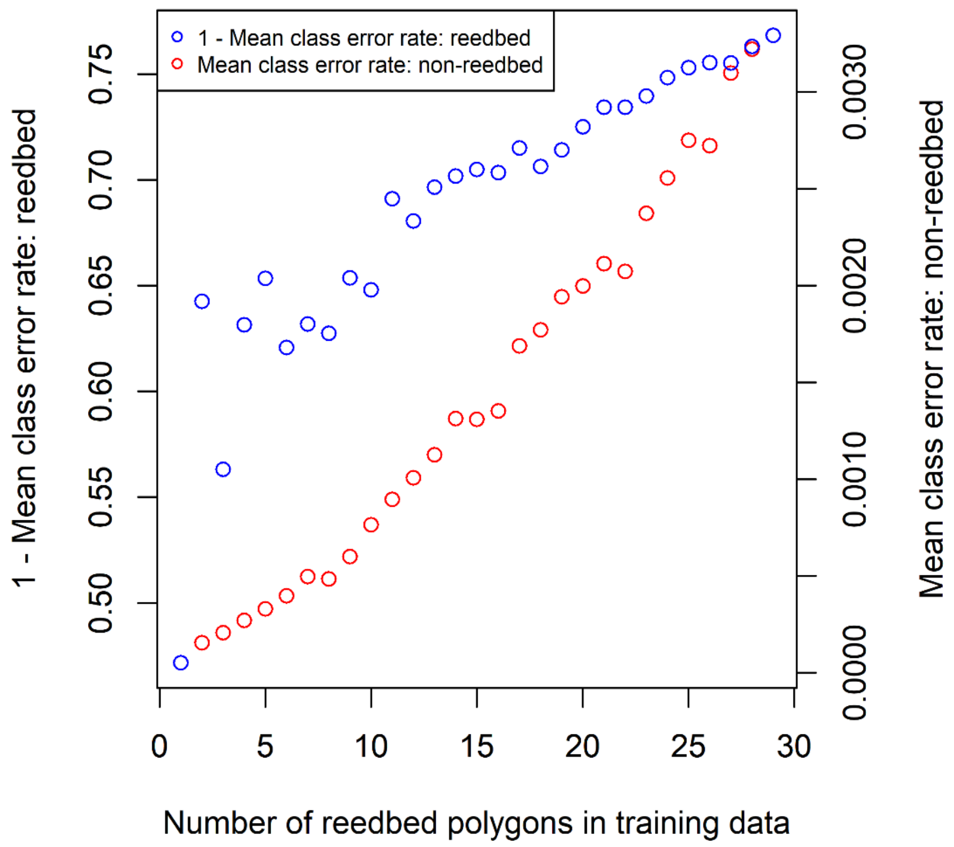

Figure S1. Effect on out-of-bag commission error rates, by class, of including additional reedbed polygons in training data. Values are average error rate over 30 random samples of included reedbed polygons.

Table S2. Confusion land cover types, HPC workflow. 'Positive' refers to the predicted or true presence of reed.

Non-reed land cover type	Negatives and true positives	False positives	False proportion of positives
**Arable**	8	51	0.864
**Conifer**	0	2	1.000
**Deciduous woodland**	4	4	0.500
**Freshwater**	3	2	0.400
**Grass farmland**	18	14	0.438
**Mixed woodland**	0	3	1.000
**Not recorded**	0	1	1.000
**Other open land cover type**	11	25	0.694
**Urban**	6	1	0.143
